# "Recreative" Reading!!

**Published:** 1901-04-27

**Authors:** 


### "Recreative" Reading!!

Our highly esteemed contemporary, the *Lancet*, was seriously exercised last week by receiving from a correspondent something which an American editor would, we imagine, describe as a cŏnundrum. The gentleman from whom it proceeded described himself as having until now possessed no time for reading, except for a "few classics," and as being desirous to repair the consequent hiatus in his intellectual equipment by the perusal of "good" novels, which he requests the editor of the *Lancet* to select and recommend. The result of the application is an article on "Recreative Reading," couched somewhat in the strain adopted by Jehoram, King of Israel, when he was invited by the King of Syria to cure Naaman of his leprosy. How, inquires our contemporary, can we advise with regard to recreative reading in the absence of any knowledge concerning the tastes and acquirements of the intending reader? The question is pertinent; but, nevertheless, the fact of the appeal having been made is one which cannot be regarded as entirely devoid of interest. The correspondent is presumably a member of the medical profession; nay, if memory does not mislead us, he even so describes himself; and yet, after the perusal of "a few classics," he desires guidance with regard to the selection of "good" novels as resources against the weariness incidental to recently-acquired leisure. The simplicity of the demand is touching, and the difficulty of complying with it is rightly described by the *Lancet* as insuperable. Is it not, nevertheless, a striking commentary upon the quality of that which the General Medical Council and the various licensing bodies accept as "education," that such a demand should be made. Every word of it veils a mystery, which, if unravelled, would probably disclose the existence of previously unsuspected depths of ignorance. What on earth are "classics," in the estimation of such an inquirer? Does he mean Hippocrates or Homer, Celsus or Gregory, Walter Scott or Pinnock? What description of novel would appear to him to fulfil the description of "good"? Novel writing, which at one time was pursued as a literary exercise, has now fallen to the condition of a trade; and perhaps the best description of a good modern novel is that it is so constructed as to enable the reader to forget the beginning by the time he reaches the middle, and to forget the middle by the time he reaches the end. It thus becomes a sort of Fortunatus's purse of literature, and can be read over and over again. But the funniest part of the whole business is the assumption by our contemporary—it does not proceed from the correspondent—that the reading is to be "recreative." Would it not, in the case of such an inquirer, more probably be "dormitative?" We remember a well-known man, speaking at a public dinner, referring to the debt of gratitude he owed to the "Short Story" writer. He said "When my brain is weary, I know no greater refreshment and relaxation before going to bed than the perusal of literature of this description, and I may say that it has been my habit for years to follow this practice." In practice the "habit" he referred to was that of falling asleep after digesting about a page of a story, and passing a considerable portion of the night asleep in his workroom instead of in bed. Again we once knew a lady, living in a country town, who went to call upon a new resident. She was ushered into a drawing-room, containing only the master of the house, sweetly slumbering in an easy chair, and was left with him by the embarrassed parlourmaid who went in search of her mistress. The sleeper, when awakened, was profuse in his apologies, but thought his condition sufficiently accounted for by saying that he "had taken up a book." Such, we imagine, would be the course of events in the case of a man presumably of more than middle age, who had reached that period of life in practically complete ignorance of literature, and destitute of the habit of attention to ideas conveyed through the medium of print. Surely, as the case of the old lady famous in story, the grandeur of the language of Johnson's "Dictionary" would be an ample compensation to such an one for any want of connectedness in the narrative. It is only when we think of the relations of such a mental state to fitness for the duties of a medical practitioner that the really appalling character of the inquiry bursts upon us in its full extent and significance. Dickens draws a telling picture of the state of a boy who is unable to read at all, and who shuffles along, ignorant of the names, and unconscious of the meanings, of the mysterious symbols so abundant over shops and at the corners of the streets; but what must be the state of a man, a member of what is called a learned profession, who in his old age requires guidance as to the direction which his reading should assume? Surely he must be conscious of at least one direction in which his knowledge is incomplete; and the best advice which could be offered to him would be to devote the remainder of his days to the upply of any such deficiency.

